# Testicular tissue transplantation: from animal models to clinical application

**DOI:** 10.1093/hropen/hoag035

**Published:** 2026-04-22

**Authors:** Ellen De Beer, Ellen Goossens

**Affiliations:** Biology of the Testis Laboratory, Research Group Genetics, Reproduction and Development, Vrije Universiteit Brussel (VUB), Brussels, Belgium; Biology of the Testis Laboratory, Research Group Genetics, Reproduction and Development, Vrije Universiteit Brussel (VUB), Brussels, Belgium

**Keywords:** fertility preservation, pre-pubertal testicular tissue, pre-pubertal boys, testicular tissue cryopreservation, testicular tissue transplantation, allotransplantation of testicular tissue, xenotransplantation of testicular tissue, autotransplantation of testicular tissue

## Abstract

**BACKGROUND:**

Every year, approximately 14,000 new cases of childhood cancer are diagnosed in Europe. Chemo- and radiotherapy have improved significantly, leading to survival rates of up to 81% in children. The growing number of childhood cancer survivors has brought increased attention to the long-term gonadotoxic effects of these treatments, with lifelong infertility being one of the most frequent side effects. Consequently, fertility preservation and restoration strategies are actively under investigation. Since spermatogenesis starts only at puberty, pre-pubertal boys cannot benefit from sperm banking. However, as spermatogonial stem cells are present from birth, the surgical retrieval and cryopreservation of pre-pubertal testicular tissue represent a promising strategy to preserve fertility in boys before undergoing gonadotoxic treatment. Pre-pubertal testicular tissue has been cryopreserved for more than 3,000 boys worldwide. At adulthood, the cryopreserved testicular tissue may be used to restore fertility, either through testicular tissue transplantation or spermatogonial stem cell transplantation (SSCT), or through *in vitro* spermatogenesis (IVS). Reviews are already available for SSCT and IVS, but an updated cross-species comparative overview of testicular tissue transplantation with emphasis on recent clinical translation is needed.

**OBJECTIVE AND RATIONALE:**

The aim of this review is to provide an overview of pre-clinical research on testicular tissue transplantation in animal models, and to highlight the initial steps of the clinical application of autologous testicular tissue transplantation.

**SEARCH METHODS:**

A comprehensive review of peer-reviewed publications on testicular tissue transplantation was performed using PubMed and Google Scholar databases. The literature search was limited to English-language studies published between 2002 (the first study on testicular tissue transplantation) and December 2025. The search was conducted using the following search terms: testicular tissue grafting, allotransplantation of testicular tissue, xenotransplantation of testicular tissue, and autotransplantation of testicular tissue. Titles and abstracts were screened for relevance. Articles not subjected to peer review were excluded. Studies were included if they involved testicular tissue grafting and reported graft outcomes, such as the most advanced germ cell stage.

**OUTCOMES:**

Animal studies have demonstrated that donor age and graft site play a crucial role in the outcome of testicular tissue transplantation. For instance, transplantation of adult testicular tissue usually led to degeneration of the seminiferous tubules, while allo-, xeno-, and autotransplantation of pre-pubertal testicular tissue successfully restores complete spermatogenesis in most of the species. In addition, homotopic engraftment generally provides the best support to restore spermatogenesis.

**LIMITATIONS, REASONS FOR CAUTION:**

As a narrative review, the risk of bias in the interpretation of findings cannot be completely eliminated.

**WIDER IMPLICATIONS:**

These findings support the clinical translation of autologous pre-pubertal testicular tissue transplantation, highlighting the potential to restore fertility and improve quality of life in men who underwent gonadotoxic treatment during childhood. Pre-pubertal testicular tissue transplantation can be considered following thorough risk assessment for patients suffering from a non-malignant disease, as well as for cancer patients with solid and non-metastatic malignancies.

**STUDY FUNDING/COMPETING INTEREST(S):**

The authors acknowledge financial support from the Vrije Universiteit Brussel (Strategic Research Program 89). The authors declare no competing interests.

WHAT DOES THIS MEAN FOR PATIENTS? Cancer treatment during childhood can lead to lifelong infertility in male survivors. As sperm production only starts at puberty, sperm banking is not an option for pre-pubertal children. However, spermatogonial stem cells (the cells that will give rise to spermatozoa from puberty onwards) are present from birth, which means that surgically retrieving and freezing pre-pubertal testicular tissue can help to preserve fertility before starting chemo- and radiotherapy. At adulthood, this pre-pubertal testicular tissue could be used to restore fertility either by transplanting the tissue or spermatogonial stem cells back into the patient, or by generating sperm cells in the lab.

Existing reviews have already covered spermatogonial stem cell transplantation and lab-based sperm generation. However, an updated overview of pre-clinical studies on testicular tissue transplantation is lacking. Therefore, this review article provides an overview of these pre-clinical studies and describes the first steps towards using testicular tissue transplantation in the clinic to restore fertility.

Animal studies have demonstrated that donor age and graft site play a crucial role in the outcome of testicular tissue transplantation. The most promising results were obtained after transplantation of pre-pubertal testicular tissue, with transplantation into the testis or scrotum being the preferred graft location. These pre-clinical findings have supported the clinical translation of autologous transplantation of human pre-pubertal testicular tissue, which is currently being evaluated in the first clinical trials.

## Introduction

### Fertility preservation in pre-pubertal boys

Every year, approximately 14,000 new cases of childhood cancer are diagnosed in Europe. As chemo- and radiotherapy have improved significantly, in children, survival rates went up to 81%, although survival depends on the cancer type. High survival rates are reported for several childhood cancers, including retinoblastoma, lymphomas, nephroblastoma, and acute lymphoblastic leukaemia (Munoz Pineiro *et al.*, 2023). The growing number of childhood cancer survivors has brought increased attention to the long-term gonadotoxic effects of these treatments, with lifelong infertility being one of the most frequent side effects (Mitchell *et al.*, 2022). Consequently, fertility preservation and restoration strategies are actively under investigation. Since spermatogenesis starts only at puberty, pre-pubertal boys are not eligible for sperm banking prior to gonadotoxic treatment. However, as spermatogonial stem cells (SSCs) are present from birth, the surgical retrieval and cryopreservation of pre-pubertal testicular tissue represent a promising strategy to preserve fertility in boys facing gonadotoxic treatment. To date, pre-pubertal testicular tissue has been cryopreserved for more than 3,000 boys worldwide (Duffin *et al.*, 2024). Currently, testicular tissue banking (TTB) is strongly recommended for young cancer patients scheduled to receive gonadotoxic treatment with a cyclophosphamide equivalent dose ≥ 4 g/m^2^. TTB is also advised for patients with non-malignant conditions (e.g. sickle-cell disease, beta-thalassaemia, bone-marrow failure) who need high-risk conditioning therapy prior to haematopoietic stem cell transplantation or bone marrow transplantation. Additionally, TTB is recommended when the estimated scattered radiation dose to the testis exceeds 1 Gy (Delgouffe *et al.*, 2022; Braye *et al.*, 2023; ESHRE FP for Boys Working Group *et al*., 2025; Safrai *et al.*, 2025).

### Fertility restoration methods

**Figure 1.**
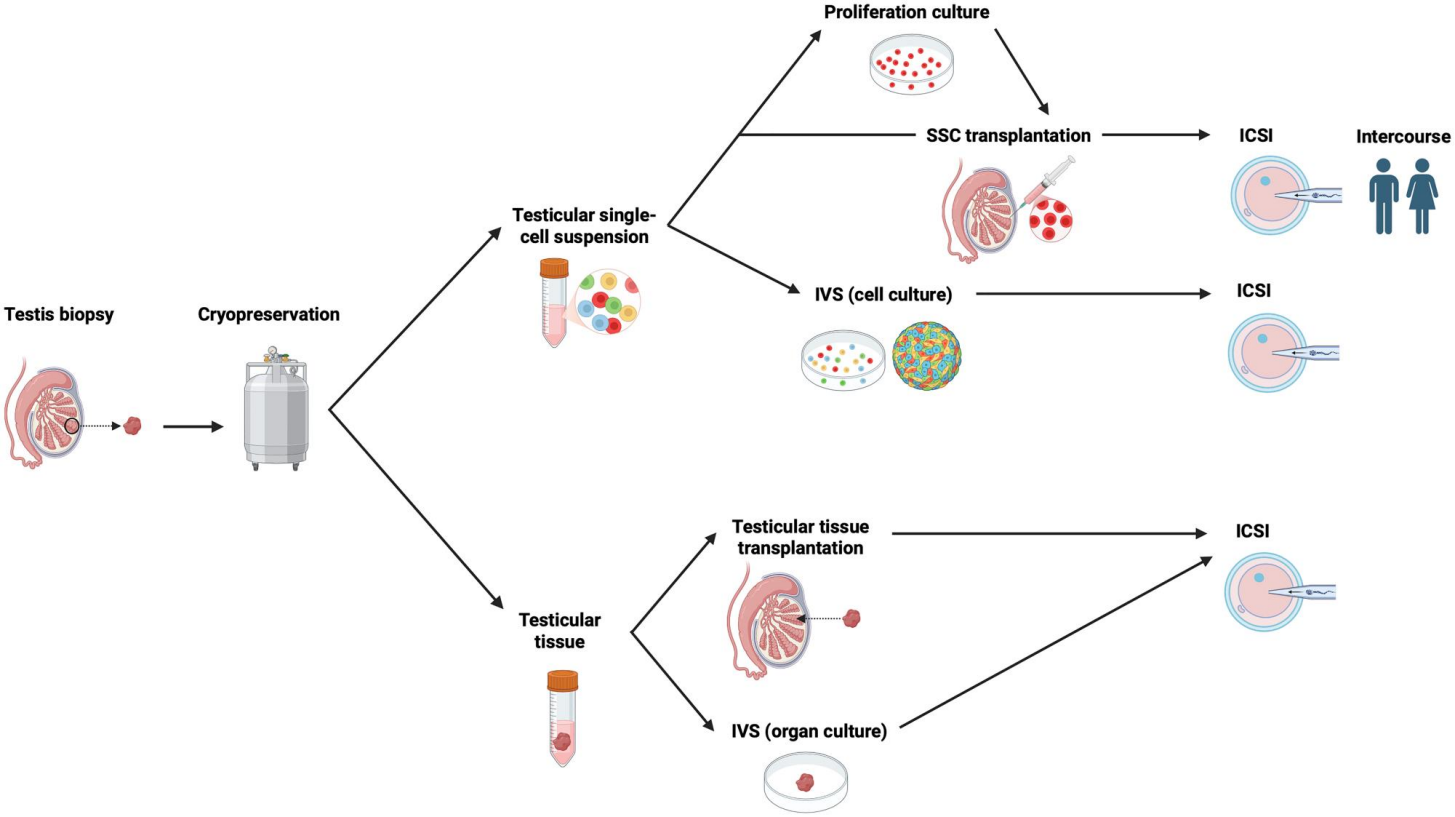
**Approaches for fertility restoration**. Testicular tissue, obtained by biopsy and subsequently cryopreserved, could be either processed into single-cell suspensions or maintained as intact tissue fragments. Spermatogonial stem cells (SSCs) could be transplanted into the testis as a suspension or within intact tissue fragments. SSCs derived from testicular cell suspensions might be proliferated *in vitro* before injection into the testis, although *in vitro* expansion remains challenging. Furthermore, testicular cell suspensions could be used for *in vitro* spermatogenesis (IVS). However, complete germ cell differentiation has not yet been established *in vitro*. In addition, intact testicular tissue can also serve as a model for IVS (organ culture). Fertility might be restored through natural intercourse or intracytoplasmic sperm injection (ICSI) after SSC transplantation, or through ICSI following testicular tissue transplantation and *in vitro* culture. Created in BioRender. Goosens, E. (2026). https://BioRender.com/zgxw66e.

Several experimental approaches are currently being investigated to restore fertility in adults who underwent gonadotoxic treatment during childhood and had their testicular tissue cryopreserved. These approaches include spermatogonial stem cell transplantation (SSCT), *in vitro* spermatogenesis (IVS), and pre-pubertal testicular tissue transplantation. Figure 1 provides a schematic overview of these fertility restoration methods.

#### Spermatogonial stem cell transplantation

SSCT was first developed in mice and has shown to be very successful, since transplanted SSCs have generated spermatozoa that were capable of producing healthy offspring (Brinster and Zimmermann, 1994). Also in rats, complete spermatogenesis has been achieved following SSCT (Ogawa *et al.*, 1999). Furthermore, SSCT in goats (Honaramooz *et al.*, 2003), sheep (Stockwell *et al.*, 2013), and pigs (Kim *et al.*, 2014) resulted in functional spermatozoa. In non-human primates, transplanted SSCs have also generated functional spermatozoa capable of supporting successful embryo development (Hermann *et al.*, 2012).

Cancer survivors diagnosed with haematological or metastatic malignancies face the risk of reintroducing malignant cells during SSCT. Two main strategies have been explored to reduce this risk. The first approach is culture-based purging, which aims to culture the testicular cell suspensions in conditions that do not support malignant cell survival. For example, acute lymphoblastic leukaemia cells could not survive for more than 26 days in a human testicular cell culture system (Sadri-Ardekani *et al.*, 2014). However, similar results could not be achieved for other cancer types (Sanou *et al.*, 2022). The second approach is marker-based selection, which aims to eliminate the risk of reintroducing malignant cells by isolating SSCs or sorting out malignant cells from the testicular cell suspension using cell-surface markers. To date, no specific marker has been found that exclusively recognizes SSCs (Sanou *et al.*, 2022), and existing markers do not allow complete removal of malignant cells from the testicular cell suspension (Geens *et al.*, 2007, 2011). However, the study by Dovey *et al.* (2013) suggests that malignant contamination can be reduced in human testicular cell suspensions through a combined use of cancer-specific and spermatogonial markers. Nevertheless, this study did not include molecular analyses such as PCR to verify the removal of malignant cells. Only morphological assessment was performed, which may be insufficient to confirm the complete elimination of malignant cells. Moreover, in pre-pubertal boys, the testicular tissue contains a limited number of SSCs. Therefore, a third approach being investigated is the *in vitro* expansion of SSCs to increase cell numbers prior to transplantation. This approach has not yet been successfully achieved using mouse SSCs due to significant losses in SSC function and number over time (Damyanova *et al.*, 2024). Moreover, Medrano *et al.* (2016) demonstrated that human SSCs show limited proliferation and largely disappear during long-term culture. However, Sadri-Ardekani *et al.* (2009, 2011) reported that their human SSC culture was successful for pre-pubertal and adult SSCs. Unfortunately, their results could not yet be reproduced. Consequently, if enough SSCs can be transplanted to fully colonize the testis, natural conception through sexual intercourse may be possible. Otherwise, conception can be achieved through testicular sperm extraction (TESE) followed by intracytoplasmic sperm injection (ICSI) (Ibtisham and Honaramooz, 2020).

An extensive review summarizing progress and challenges in the field of SSCT has been published (Gul *et al.*, 2020).

#### In vitro spermatogenesis

IVS approaches include monolayer cell cultures, 3-dimensional (3D) cell cultures, and organ cultures. It has been shown that germ cell survival within monolayer culture systems is limited (von Kopylow *et al.*, 2018). Several 3D culture models have been developed to support spermatogenesis. For instance, murine testicular organoids have demonstrated long-term germ cell survival and post-meiotic differentiation (Richer *et al.*, 2021). Nevertheless, achieving complete spermatogenesis in testicular organoids derived from other species and humans remains challenging (Stukenborg, 2025). 3D bioprinting of mouse testicular cells resulted in the formation of elongating spermatids (Baert *et al.*, 2019) and human SSCs generated sporadic round spermatids when cultured in a 3D Matrigel^®^ system (Sun *et al.*, 2018). Organ cultures have been shown to support complete spermatogenesis, resulting in healthy offspring in mice (Sato *et al.*, 2013). Complete spermatogenesis has also been achieved in organ cultures from rat and bovine testicular tissue (Kim *et al.*, 2015; Liu *et al.*, 2016a). Organ culture of pre-pubertal human testicular tissue supports *in vitro* formation of a partial blood–testis barrier, somatic cell maturation, and the production of haploid germ cells (de Michele *et al.*, 2018a,b). However, human testicular tissue cultures are hindered by the limited availability of pre-pubertal donor tissue. For research purposes, human foetal testis tissue has served as an alternative to pre-pubertal tissue. These tissues progressed to round spermatids capable of fertilizing oocytes and supporting early embryonic development (Kulibin and Malolina, 2023).

Obviously, IVS systems will necessitate the use of ICSI to achieve fertilization.

Comprehensive reviews on IVS have been published (Richer *et al.*, 2020; Nguyen *et al.*, 2024).

#### Testicular tissue transplantation

In animal models, testicular tissue fragments from donors of various ages have been transplanted either back to its original site in the scrotum or testis (homotopic grafting), or to a different location in the body (ectopic grafting), with tissues transplanted either fresh or frozen-thawed. Overall, allo-, xeno-, and autotransplantation of pre-pubertal testicular tissue has been successful in most of the species, leading to the restoration of complete spermatogenesis.

Testicular tissue transplantation using tissue from patients with a history of haematological malignancies, such as leukaemia, or metastatic cancers carries a substantial risk of reintroducing malignant cells due to the possible presence of malignant cells in the testicular tissue. Therefore, autologous testicular tissue transplantation may be proposed to patients suffering from a non-malignant disease, as well as for cancer patients with solid or non-metastatic tumours following thorough risk assessment.

In order to induce a pregnancy, ICSI will be required in case of ectopic transplantations, but also after intratesticular grafting because spermatozoa produced in the transplanted tissue will probably not be able to reach the rete testis due to the lack of a direct connection (Safrai *et al.*, 2025).

**Figure 2.**
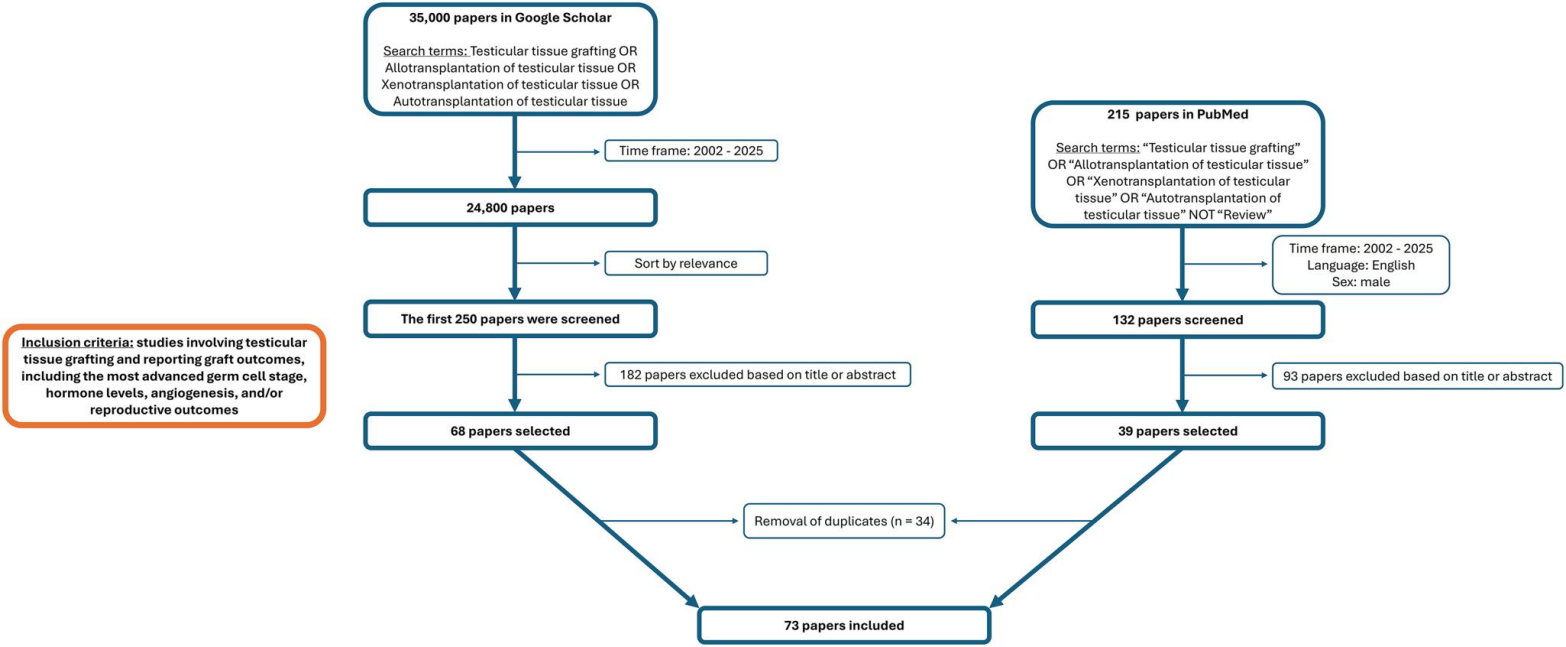
**Flowchart of article selection process**. A systematic search was conducted in Google Scholar and PubMed to identify studies investigating testicular tissue grafting. Studies involving testicular tissue grafting and reporting grafting outcomes, including the most advanced germ cell stage, hormone levels, angiogenesis, and/or reproductive outcomes, were included. In Google Scholar, a total of 24 800 records were initially obtained using the search terms: testicular tissue grafting or allotransplantation of testicular tissue or xenotransplantation of testicular tissue or autotransplantation of testicular tissue, restricted to the time frame 2002–2025. Following sorting by relevance, the first 250 articles were screened based on title and abstract, with 68 studies meeting the predefined inclusion criteria. In PubMed, 215 records were obtained using the same search terms and time frame, with exclusion of reviews. After restricting the language to English and limiting the sex to males, 132 records were available. These articles were screened based on title and abstract, with 39 studies meeting the predefined inclusion criteria. After removing 34 duplicates across the two databases, a total of 73 studies were included in this review article.

Reviews on testicular tissue transplantation date back several years (Arregui and Dobrinski, 2014; Kilcoyne and Mitchell, 2019). In this review paper, we provide an updated cross-species comparative overview of testicular tissue transplantation with emphasis on recent clinical translation. Figure 2 shows a flowchart of the article selection process for this review.

## Allotransplantation of testicular tissue

Allogeneic testicular tissue transplantation has been conducted in medaka, mice, rats, and rabbits. An overview of these findings is shown in Table 1.

**Table 1.** Allotransplantation of testicular tissue in various species.

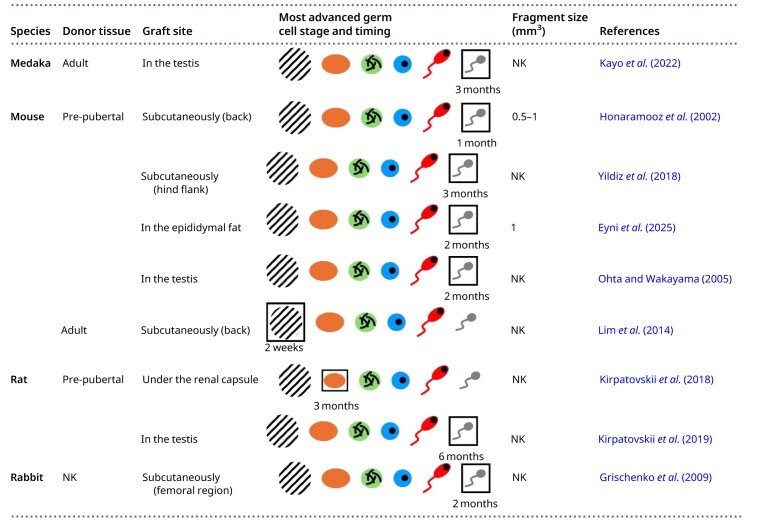

Hatched areas indicate graft degeneration, orange represents spermatogonia, green represents spermatocytes, blue represents round spermatids, red represents elongating spermatids, and grey represents spermatozoa. The most advanced germ cell stage is outlined. NK, not known.

### Medaka

Kayo *et al.* (2022) transplanted testicular tissue from adult donor medaka into partially castrated adult recipients, specifically into the remaining part of the recipient testes. These allografts survived for at least 3 months and produced functional sperm that supported donor-derived offspring through natural mating. These results indicate that sperm from the transplanted testicular tissue was released into the efferent duct, suggesting functional integration of the graft within the host testicular tissue.

### Mouse

Allografting of pre-pubertal mouse testicular tissue fragments was performed ectopically under the back skin of castrated immunodeficient mice (Honaramooz *et al.*, 2002; Geens *et al.*, 2006; Yu *et al.*, 2006). Due to the high variability of serum testosterone concentrations in mice, endocrine function was assessed indirectly by measuring the weight of the seminal vesicles. The observed increase in seminal vesicle weight in these recipient mice indicated elevated testosterone levels (Honaramooz *et al.*, 2002). From 1 month post-transplantation onwards, complete spermatogenesis was observed in these ectopic allografts (Honaramooz *et al.*, 2002; Yu *et al.*, 2006). Similarly, Geens *et al.* (2006) observed complete spermatogenesis in one-third of such allografts. Furthermore, spermatozoa retrieved from these allografts successfully fertilized oocytes via ICSI, leading to embryos that developed normally to the foetal stage (Honaramooz *et al.*, 2002). Moreover, ectopic allografting of frozen-thawed pre-pubertal mouse testicular tissue resulted in complete spermatogenesis within 4 months (Milazzo *et al.*, 2010). Complete spermatogenesis was also observed 3 months after subcutaneous allografting of both fresh and frozen-thawed pre-pubertal mouse testicular tissue into the hind flank. Remarkably, grafts preserved using slow freezing showed higher testicular tissue survival compared to vitrified grafts (Yildiz *et al.*, 2018). Complete spermatogenesis was also observed after 2 months when pre-pubertal testicular tissue was allografted into the epididymal fat of castrated immunodeficient mice (Eyni *et al.*, 2025), and within 2–4 months when allografted into the testes (Shinohara *et al.*, 2002; Ohta and Wakayama, 2005; Van Saen *et al.*, 2009). Spermatozoa retrieved from these intratesticular allografts were capable of producing healthy offspring via ICSI (Shinohara *et al.*, 2002; Ohta and Wakayama, 2005).

In contrast, ectopic allografting of adult mouse testicular tissue fragments under the back skin resulted in graft degeneration. Graft degeneration started within 2 weeks after grafting, with clear histological damage observed around 1 month (Geens *et al.*, 2006; Arregui *et al.*, 2012; Lim *et al.*, 2014).

### Rat

Kirpatovskii *et al.* (2018) conducted the first study in which fresh and frozen-thawed testicular tissue from pre-pubertal rats was transplanted under the renal capsule. Serum testosterone levels increased significantly within 1 month after transplantation, continued to rise up to 3 months, and stabilized at ∼50% of normal testosterone levels. However, histological analysis showed that spermatogenesis was not initiated, with germ cells remaining at the spermatogonial stage. Factors that may contribute to the failure to initiate spermatogenesis include low testosterone levels and persistent immune responses, as evidenced by the formation of a connective tissue capsule around the graft and eosinophil infiltration of the stromal tissue. In their next study, the hosts were experimentally induced with abdominal cryptorchidism to disrupt spermatogenesis, and pre-pubertal testicular tissue was transplanted into the testes (Kirpatovskii *et al.*, 2019). As a result, serum testosterone levels normalized within 2 months, and complete spermatogenesis was observed by 6 months post-transplantation. Furthermore, Deng *et al.* (2020) grafted both fresh and frozen-thawed pre-pubertal rat testicular tissue into the testes of immunosuppressed recipients. One month after transplantation, spermatocytes were the most advanced germ cell stage observed in both fresh and frozen-thawed allografts, but longer follow-up data were not available.

### Rabbit

Grischenko *et al.* (2009) performed allotransplantation of frozen-thawed testicular tissue fragments from rabbits into recipients that had undergone 2 months of sexual abstinence, resulting in testicular hypofunction. The testicular fragments were encapsulated in an amniotic membrane to form a ‘sac’, which is impermeable to immunocompetent cells, and were transplanted subcutaneously on the external side of the femoral region. Two months after grafting, complete spermatogenesis was observed.

## Xenotransplantation of testicular tissue into mice

All described xenotransplantations of testicular tissue used donor tissue from different species transplanted into immunodeficient mice. Xenotransplantation of testicular tissue into mice has been established for a range of donor species, including rodents, lagomorphs, domestic animals, large wild mammals, non-human primates, and humans. Ectopic xenotransplantation was performed subcutaneously under the back skin, while homotopic xenotransplantation was carried out under the scrotal skin or in the testes. Except for intratesticular transplantations, immunodeficient host mice were castrated, unless otherwise indicated. An overview of these findings is shown in Table 2.

**Table 2.** Xenotransplantation of testicular tissue from various species into mice.

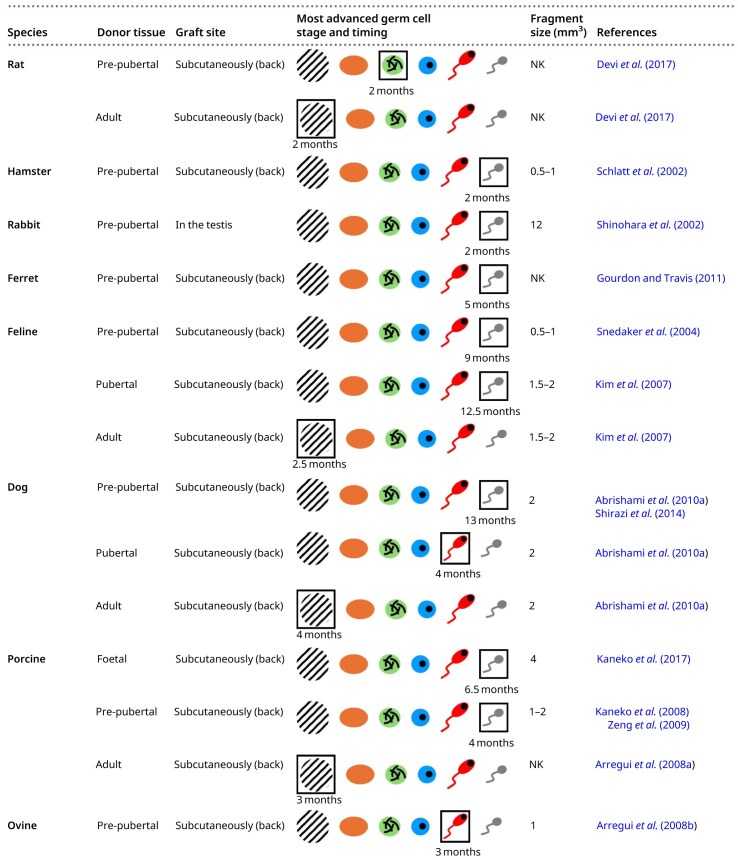
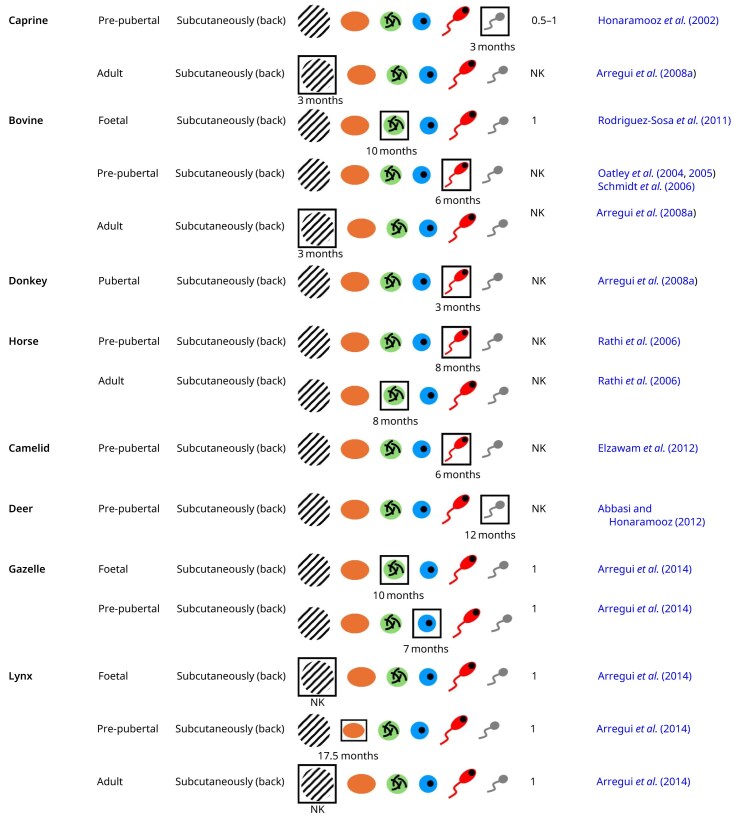
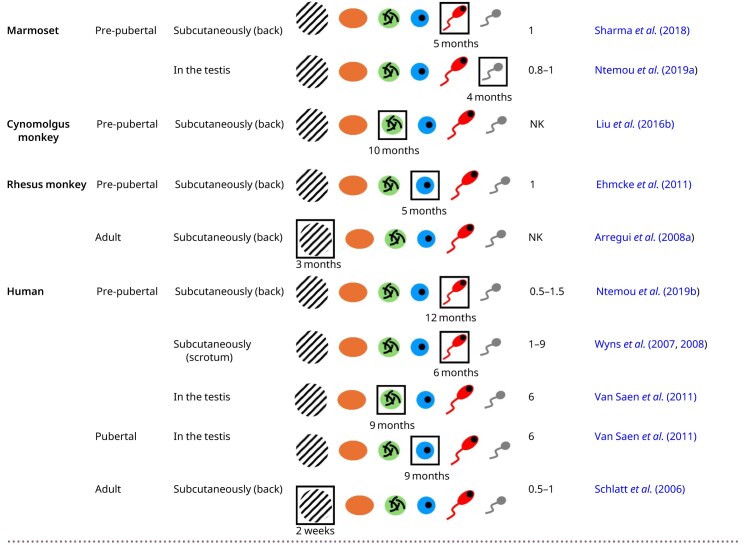

Hatched areas indicate graft degeneration, orange represents spermatogonia, green represents spermatocytes, blue represents round spermatids, red represents elongating spermatids, and grey represents spermatozoa. The most advanced germ cell stage is outlined. NK, not known.

### Rodents and lagomorphs

#### Rat

Devi *et al.* (2017) demonstrated that pre-pubertal ectopic xenografts derived from rats and transplanted into mice were capable of initiating angiogenesis, as evidenced by the presence of angiogenesis-related signalling proteins at 1 and 2 weeks post-grafting. Schlatt *et al.* (2010) further confirmed that these xenografts were connected to the subcutaneous circulation through both donor-derived capillary outgrowth and host vessel formation. Furthermore, recipient mice grafted with rat pre-pubertal testicular tissue maintained normal serum LH and testosterone levels, but showed significantly elevated FSH levels, likely due to the absence of FSH receptors in the xenografts (Goel and Minami, 2019). At 1 and 2 months post-grafting, these pre-pubertal ectopic donor grafts showed partial spermatogenesis, with pachytene spermatocytes representing the most advanced germ cell stage observed. In addition, at 2 months post-grafting, pre-pubertal xenografts showed a significant increase in proliferating germ cells alongside a decrease in proliferating Sertoli cells, although proliferating Sertoli cells were still present. The presence of these proliferating Sertoli cells indicates delayed maturation, which may explain the incomplete spermatogenesis (Devi *et al.*, 2017). Pre-pubertal grafts also demonstrated dysregulated expression of key genes encoding Sertoli cell transcription factors (Goel and Minami, 2019).

Two months after ectopic grafting of adult rat testicular tissue, xenografts showed severe degeneration of the seminiferous tubules, lack of proliferating germ cells, and only a few remaining spermatozoa. This was probably due to a suboptimal graft vascularization, since, at 1 and 2 weeks post-grafting, these adult xenografts showed a reduction of angiogenesis-related signalling proteins (Devi *et al.*, 2017).

#### Hamster

Schlatt *et al.* (2002) performed ectopic xenotransplantation into mice of pre-pubertal hamster testicular tissue fragments, using either fresh or frozen-thawed tissue. Notably, assessment of seminal vesicle weight indicated that fresh grafts restored normal testosterone levels, while frozen-thawed grafts showed a reduction. However, at 2 months post-grafting, spermatozoa were present in more than 25% of the seminiferous tubules within fresh and frozen-thawed xenografts. These findings demonstrate that although grafting frozen-thawed testicular tissue restores testosterone production less effectively, the tissue still maintains the capacity to support spermatogenesis.

#### Rabbit

Shinohara *et al.* (2002) carried out xenotransplantation of frozen-thawed pre-pubertal rabbit testicular tissue fragments into the mouse testes. Complete spermatogenesis was restored within 2 months after transplantation, resulting in spermatozoa able to give rise to the birth of healthy rabbit offspring.

#### Cross-species synthesis

Ectopic xenografting of pre-pubertal testicular tissue resulted in meiotic arrest (rat) or in complete spermatogenesis (hamster). Homotopic xenotransplantation of pre-pubertal testicular tissue yielded functional spermatozoa capable of producing offspring (rabbit).

Ectopic transplantation of adult testicular tissue led to degeneration of the seminiferous tubules (rat).

### Domestic animals

#### Ferret

Gourdon and Travis (2011) performed ectopic xenotransplantation of testicular tissue from pre-pubertal ferrets. The seminal vesicle weight indicated that these xenografts secreted bioactive testosterone. At 2.5 months post-transplantation, primary spermatocytes were the most advanced germ cell stage observed. At 5 months post-grafting, spermatozoa were detected in 14% of the xenografts, increasing to 30% at 6 months. Remarkably, only 8% of the xenografts contained spermatozoa at 7.5 months post-transplantation, indicating that grafts may have a limited functional lifespan.

#### Feline

In ectopic xenografts of feline pre-pubertal testicular tissue, pachytene spermatocytes appeared around 4.5 months post-grafting, followed by the first detection of round spermatids at 5 months. Spermatozoa were first detected around 9 months post-grafting (Snedaker *et al.*, 2004).

Ectopic xenotransplantation of pubertal testicular tissue also resulted in the production of spermatozoa, which were first detectable 1 year after grafting (Kim *et al.*, 2007).

In contrast, ectopic xenografts obtained from adult donors showed degeneration of the seminiferous tubules (Kim *et al.*, 2007).

#### Dog

At 13 months post-grafting, ectopic xenografts of pre-pubertal dog testicular tissue showed complete spermatogenesis (Abrishami *et al.*, 2010a). Spermatozoa from these xenografts were capable of inducing oocyte activation and promoting male pronucleus formation (Shirazi *et al.*, 2014).

At 4 months post-grafting, ectopic xenografts from an early pubertal (4.5-month-old) donor revealed spermatogenic development up to the formation of elongating spermatids. However, by 8 months post-grafting, these xenografts contained no differentiated germ cells anymore. Xenografts from an older pubertal donor (5.5-month-old) already showed degeneration in more than 60% of seminiferous tubules at 4 months post-grafting. By 8 months post-grafting, spermatocytes were the pre-dominant germ cell type, with only 2–3% of tubules containing round spermatids (Abrishami *et al.*, 2010a).

Ectopic xenografts from adult donors showed prominent tubular degeneration by 4 months post-grafting, progressing to complete degeneration by 8 months (Abrishami *et al.*, 2010a).

#### Porcine

After ectopic xenografting of frozen-thawed testicular tissue from porcine foetuses, the serum levels of both inhibin and testosterone were elevated in the recipient mice. Spermatozoa were detected between 6.5 and 15 months post-transplantation. At 6.5 months, 10–20% of the tubules contained germ cells that had progressed beyond the spermatogonial stage, while less than 5% showed elongating spermatids or spermatozoa. Between 13 and 15 months post-transplantation, tubule maturation had progressed significantly, with over 45% of tubules containing elongating spermatids or spermatozoa. However, no piglets were born after ICSI and subsequent embryo transfer (Kaneko *et al.*, 2017).

One month after ectopic transplantation of fresh testicular tissue fragments from pre-pubertal pigs, serum inhibin levels were significantly elevated. Remarkably, serum inhibin levels continued to rise beyond 3 months, reaching values higher than those observed in non-castrated mice. Elevated inhibin levels may directly influence spermatogenic activity and contribute to the suppression of FSH secretion. At 2 months post-transplantation, serum FSH levels in recipient mice declined, likely reflecting both the suppressive effect of inhibin and the establishment of a functionally active feedback loop between the mouse hypothalamic–pituitary–gonadal (HPG) axis and the grafted testicular tissue. Furthermore, testosterone levels increased from 3 months onwards (Kaneko *et al.*, 2008). Spermatocytes were first observed within 2 months, with spermatids appearing by 3 months. At 10 months post-grafting, spermatids were present in 67% of the xenografts (Watanabe *et al.*, 2009). Spermatozoa were first detected at 4 months and continued to be present until at least 10 months post-grafting (Honaramooz *et al.*, 2008; Kaneko *et al.*, 2008; Watanabe *et al.*, 2009; Abbasi and Honaramooz, 2011a; Campos-Junior *et al.*, 2014). Spermatozoa recovered from these fresh pre-pubertal xenografts were able to fertilize oocytes and produce diploid embryos expressing the paternally imprinted gene neuronatin, confirming their epigenetic competence (Honaramooz *et al.*, 2008; Campos-Junior *et al.*, 2014). Following ICSI, 2 of the 23 recipient gilts gave birth. The first gilt delivered a single healthy piglet, which produced motile spermatozoa at around 8 months of age. The second gilt gave birth to five piglets, all of which showed normal development by the pre-pubertal age of 4 months (Nakai *et al.*, 2010). Ectopic grafting of frozen-thawed pre-pubertal porcine testicular tissue fragments resulted in hormone levels comparable to those observed in fresh grafts (Honaramooz *et al.*, 2002). Furthermore, complete spermatogenesis was observed in these xenografts between 4 and 9 months post-grafting (Honaramooz *et al.*, 2002; Zeng *et al.*, 2009; Abrishami *et al.*, 2010b). Kaneko *et al.* (2013) retrieved spermatozoa from such xenografts leading to the birth of healthy offspring.

In contrast, complete degeneration of seminiferous tubules was observed 3 months after ectopic xenografting of adult porcine testicular tissue fragments (Arregui *et al.*, 2008a).

#### Ovine

Fragments of pre-pubertal ovine testicular tissue, either fresh, slow-frozen, or vitrified, were ectopically xenografted (Zeng *et al.*, 2005; Arregui *et al.*, 2008b; Pukazhenthi *et al.*, 2015). One month after grafting fresh tissue, spermatogonia were present in ∼43% of the tubules, but had not yet started differentiation. At 2 months post-grafting, the percentage of tubules containing spermatogonia had increased significantly to 70%, with ∼32% of tubules containing pachytene spermatocytes. At 3 months after grafting, elongating spermatids were present in ∼5% of tubules, which further increased to around 19% by 4 months post-grafting (Zeng *et al.*, 2005; Arregui *et al.*, 2008b). The most advanced germ cells identified at 1 h, 11 days, and 22 days were pre-leptotene/leptotene spermatocytes, pachytene primary spermatocytes, and elongating spermatids, respectively, which indicates that the timing of germ cell development is well preserved in ovine xenografts (Zeng *et al.*, 2005). At 4 months post-grafting, tissue that had been slow-frozen supported the development of elongating spermatids, while tissue that had been vitrified resulted only in development up to the primary spermatocyte stage (Pukazhenthi *et al.*, 2015).

#### Caprine

Complete spermatogenesis was achieved 3 months after ectopic xenografting of pre-pubertal goat testicular tissue fragments. Large numbers of spermatozoa were recovered from these xenografts and were subsequently used to fertilize goat oocytes via ICSI, leading to embryos showing normal development (Honaramooz *et al.*, 2002).

In contrast, ectopic xenotransplantation of adult goat testicular tissue fragments led to degeneration of the seminiferous tubules within 3 months post-transplantation, with no evidence of spermatogenic recovery or sperm production (Arregui *et al.*, 2008a).

#### Bovine

Ten months after ectopic grafting of foetal bovine testicular tissue, the xenografts showed testosterone production, as evidenced by increased seminal vesicle weight in recipient mice. Pachytene spermatocytes were the most advanced germ cell stage present in these xenografts. The lack of more mature germ cells was likely due to incomplete Sertoli cell maturation, as indicated by a progressive but incomplete loss of anti-Müllerian hormone expression and weak androgen receptor expression (Rodriguez‐Sosa *et al.*, 2011).

After ectopic grafting of fresh pre-pubertal bovine testicular tissue, testosterone levels in recipient mice remained within the normal range from 1 to 6 months post-grafting (Honaramooz *et al.*, 2004a; Oatley *et al.*, 2004, 2005; Schmidt *et al.*, 2006). However, germ cell numbers declined significantly between 1 and 2 months after transplantation, although this decrease was not due to germ cell apoptosis. After 2 months, germ cell numbers started to rise. Pachytene spermatocytes were first observed in xenografts collected between 3 and 4 months post-grafting, but no further progression was found thereafter (Rathi *et al.*, 2005). Two studies confirmed this maturation arrest (Honaramooz *et al.*, 2004a; Reddy *et al.*, 2012), although other studies did observe elongating spermatids between 6 and 16 months following transplantation of pre-pubertal bovine testicular tissue (Oatley *et al.*, 2004, 2005; Rathi *et al.*, 2005; Schmidt *et al.*, 2006; Huang *et al.*, 2008; Abbasi and Honaramooz, 2011b). Furthermore, 1 month after ectopic transplantation of frozen-thawed pre-pubertal tissue, vascularization was observed around the grafts, but no further follow-up of graft development was performed (Zhao *et al.*, 2025).

In contrast, 3 months after ectopic grafting, adult bovine testicular tissue xenografts showed complete degeneration of the tubules (Arregui *et al.*, 2008a).

#### Donkey

Arregui *et al.* (2008a) demonstrated that ectopic xenografting of testicular tissue fragments from pubertal donkeys supported spermatogenesis. Specifically, elongating spermatids were detected in the grafts between 3 and 6 months post-transplantation.

#### Horse

Rathi *et al.* (2006) performed ectopic xenotransplantation using testicular tissue fragments from foals. The increase in seminal vesicle weight suggested that these xenografts secreted testosterone. At 8 months post-grafting, elongating spermatids represented the most advanced germ cells.

After ectopic transplantation of adult testicular tissue, there was evidence of testosterone secretion. Nevertheless, pachytene spermatocytes were the most advanced germ cells observed at 4 and 8 months post-grafting (Rathi *et al.*, 2006).

#### Camelid

Ectopic xenotransplantation of pre-pubertal alpaca testicular tissue resulted in testosterone production and the appearance of elongating spermatids after 6 months (Elzawam *et al.*, 2012).

#### Cross-species synthesis

Ectopic xenografting of foetal testicular tissue resulted in meiotic arrest (bovine) or complete spermatogenesis (porcine).

Ectopic transplantation of pre-pubertal testicular tissue supported spermatogenesis up to elongating spermatids (ovine, bovine, equine, and alpaca) or spermatozoa (ferret, feline, dog, porcine, and goat).

Ectopic xenografts of pubertal testicular tissue yielded elongating spermatids (dogs and donkeys) or spermatozoa (feline).

Degeneration of the seminiferous tubules was observed following ectopic xenotransplantation of adult testicular tissue.

### Large wild mammals

#### Deer

Abbasi and Honaramooz (2012) performed xenotransplantation of fresh pre-pubertal testicular tissue fragments from white-tailed deer under the back skin of castrated immunodeficient mice. These xenografts showed spermatocytes by 6 months post-grafting, progressed to round and elongating spermatids by 8 months, and produced spermatozoa by 12 months post-grafting. Pothana *et al.* (2015) xenografted fragments of frozen-thawed pre-pubertal testicular tissue from Indian spotted mouse deer under the back skin of non-castrated immunodeficient mice. At 6 months post-grafting, pachytene spermatocytes were the most advanced germ cell stage present.

#### Gazelle

After ectopic xenografting of foetal gazelle testicular tissue fragments, the seminal vesicle weight started to increase 3 months post-grafting, thereby indicating the onset of testosterone production. Furthermore, pachytene spermatocytes were the most advanced germ cell stage after 10 months (Arregui *et al.*, 2014).

Ectopic grafting of fresh and frozen-thawed pre-pubertal testicular tissue fragments resulted in elevated testosterone production from 3  months post-grafting onwards. This may explain why no differentiated germ cells were detected until 3 months after transplantation. At 4 months post-grafting, 7% of tubules contained spermatocytes and 1% contained round spermatids. Similar findings were observed at 7 months post-grafting (Arregui *et al.*, 2014).

#### Lynx

Degeneration of the seminiferous tubules was observed in ectopic xenografts derived from frozen-thawed foetal lynx testicular tissue (Arregui *et al.*, 2014).

In frozen-thawed pre-pubertal ectopic xenografts, the elevated seminal vesicle weight observed 10 months after grafting indicated ongoing testosterone secretion. Furthermore, spermatogonia persisted for at least 17.5 months, with the proportion of tubules containing spermatogonia increasing between 7 and 8 months post-grafting. However, differentiated germ cells were absent at all time points (Arregui *et al.*, 2014).

Ectopic xenografts derived from fresh adult donor tissue showed degeneration of the seminiferous tubules (Arregui *et al.*, 2014).

#### Cross-species synthesis

Ectopic xenografts of foetal testicular tissue showed degeneration of the seminiferous tubules (lynx) or meiotic arrest (gazelle).

Ectopic xenografts of pre-pubertal testicular tissue resulted in spermatogonial survival for at least 17.5 months (lynx), the appearance of round spermatids (gazelle) or the production of spermatozoa (deer).

Degeneration of the seminiferous tubules was observed following ectopic xenotransplantation of adult testicular tissue (lynx).

### Non-human primates

#### Marmoset

Pre-pubertal testicular tissue fragments from marmosets were grafted into mice under the back skin (either fresh or frozen-thawed) and into the testes (using fresh tissue only) (Schlatt *et al.*, 2002; Sharma *et al.*, 2018; Ntemou *et al.*, 2019a). Testosterone levels remained low in mice receiving ectopic xenografts (Schlatt *et al.*, 2002). At 4 months post-grafting, spermatocytes in these ectopic xenografts did not progress through meiosis (Ntemou *et al.*, 2019a). After 5 months, early spermatids were sporadically observed (Sharma *et al.*, 2018), but none of these grafts survived beyond 9 months (Ntemou *et al.*, 2019a). The common marmoset carries a deletion of exon 10 in the LH receptor gene, which limits its responsiveness to LH. As a result, reduced LH responsiveness limits testosterone production in the graft, increasing the need for high testosterone levels around the graft to support complete spermatogenesis (Wistuba *et al.*, 2004). In both rats and humans, it is shown that the total testosterone concentration in serum is far lower than in the testis. This may be explained by the presence of androgen-binding proteins within the testis, which enable it to function as a reservoir for testosterone (Jarow and Zirkin, 2005). Therefore, the meiotic arrest in these xenografts could be attributed to insufficient testosterone levels caused by the ectopic location of the grafts, where Leydig cells receive insufficient stimulation, resulting in reduced testosterone production and failure of Sertoli cell maturation. Moreover, the testis maintains a lower temperature, suggesting that the elevated temperature at the ectopic transplantation site may contribute to spermatogenic arrest. In contrast, homotopic grafts are exposed to higher testosterone levels, which compensates for insufficient LH receptor activation and promotes progression of germ cell differentiation (Jarow and Zirkin, 2005; Sharma *et al.*, 2018). As a result, complete spermatogenesis was achieved from 4 months onwards following intratesticular xenografting and this increased further by 9 months (Ntemou *et al.*, 2019a).

#### Cynomolgus monkey

Liu *et al.* (2016b) ectopically xenotransplanted fragments of pre-pubertal testicular tissue from cynomolgus monkeys. Normal vascularization was established before 3 months post-transplantation and was maintained throughout the grafting period. By 3 months post-grafting, spermatogonia were the most advanced germ cells observed, but between 10 and 17 months post-grafting, spermatozoa could be isolated and used for ICSI, which ultimately led to the birth of six healthy monkeys.

#### Rhesus monkey

Ectopic grafting of pre-pubertal rhesus monkey testicular tissue restored testosterone secretion, as evidenced by a significant increase in seminal vesicle weight in the recipient mice. At 2 months post-grafting, spermatogonia were the only germ cells observed. At 4 months post-grafting, spermatocytes and round spermatids were present. Spermatozoa appeared between 5 and 7 months after grafting (Honaramooz *et al.*, 2004b; Ehmcke *et al.*, 2011). Spermatozoa retrieved from these pre-pubertal xenografts showed typical rhesus testicular sperm morphology, with more than 80% viability and up to 10% motility. Following ICSI, several oocytes underwent cleavage, and all developed to the morula stage. Some embryos progressed further to the blastocyst stage, demonstrating the fertilization competence of these spermatozoa (Honaramooz *et al.*, 2004b).

Three months after ectopic grafting of adult testicular tissue, xenografts showed degeneration of seminiferous tubules (Arregui *et al.*, 2008a).

#### Cross-species synthesis

Ectopic xenografts of pre-pubertal testicular tissue produced functional spermatozoa (cynomolgus and rhesus monkeys). In marmoset, ectopic xenografts contained round spermatids as the most advanced germ cell stage, while homotopic marmoset xenografts demonstrated complete spermatogenesis.

Degeneration of the seminiferous tubules was observed following ectopic xenotransplantation of adult testicular tissue (rhesus monkey).

### Human

Fresh and frozen-thawed testicular tissue fragments from pre-pubertal boys were ectopically xenografted into mice. Goossens *et al.* (2008) reported that SSCs survived for at least 9 months in frozen-thawed xenografts. Furthermore, pachytene spermatocytes were the most advanced germ cell stage observed 9–12 months after ectopic grafting fresh or frozen-thawed testicular tissue (Sato *et al.*, 2010; Ntemou *et al.*, 2019b). Additionally, frozen-thawed testicular tissue fragments from pre-pubertal boys were xenografted into mice under the scrotal skin. Six months later, germ cell development progressed up to the pachytene stage (Wyns *et al.*, 2007, 2008). Furthermore, testicular tissue fragments from pre-pubertal boys were grafted, either fresh or frozen-thawed, into the mouse testes. At 9 months post-grafting, the intratesticular xenografts showed no germ cell differentiation (Van Saen *et al.*, 2011). This arrest may be due to the insufficient stimulation of human Leydig and Sertoli cells by mouse LH and FSH, which reflects a known limitation of the mouse xenograft model in supporting human tissue maturation. Consequently, human Leydig cells may have produced inadequate levels of testosterone, which is essential for Sertoli cell maturation and the initiation of spermatogenesis (Hutka *et al.*, 2020).

Testicular tissue from pubertal boys, either fresh or frozen-thawed, was grafted into the testes. Nine months after grafting, xenografts showed differentiation up to the stage of secondary spermatocytes (Van Saen *et al.*, 2011).

Following ectopic xenografting of adult donor tissue, reduced seminal vesicle weight in recipient mice suggested low testosterone production. Within 2 weeks post-grafting, xenografts from adult donors showed degeneration of the seminiferous tubules (Schlatt *et al.*, 2006).

## Autotransplantation of testicular tissue

Autotransplantation of testicular tissue has been demonstrated in trout, rats, non-human primates, and humans. An overview of these findings is shown in Table 3.

**Table 3.** Autotransplantation of testicular tissue in various species.

Species	Donor tissue	Graft site	Most advanced germ cell stage and timing	Fragment size (mm³)	References
**Trout**	Pre-pubertal	Subcutaneously (back)	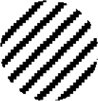 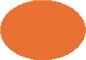 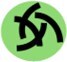 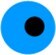 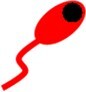 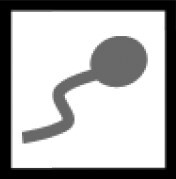 5 months	NK	Hayashi *et al.* (2018)
**Rat**	Pre-pubertal	Subcutaneously (scrotum)	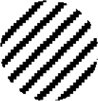 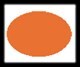 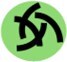 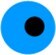 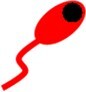 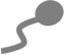 1 month	1–27	Wang *et al.* (2022)
**Marmoset**	Pre-pubertal	Subcutaneously (back)	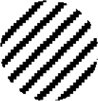 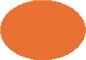 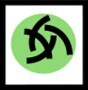 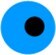 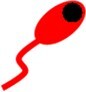 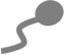 17 months	0.5–1	Wistuba *et al.* (2006)
		Subcutaneously (scrotum)	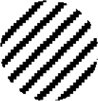 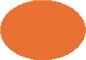 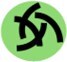 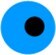 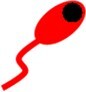 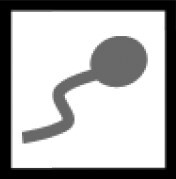 NK	NK	Luetjens *et al.* (2008)
	Adult	Subcutaneously (back)	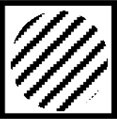 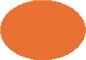 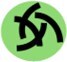 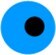 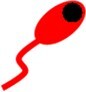 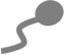 NK	NK	Luetjens *et al.* (2008)
**Rhesus monkey**	Pre-pubertal	Subcutaneously (shoulder)	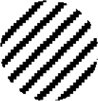 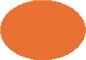 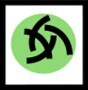 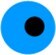 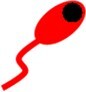 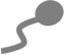 5 months	1	Jahnukainen *et al.* (2012)
		Subcutaneously (back)	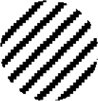 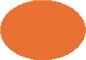 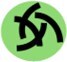 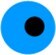 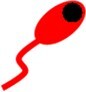 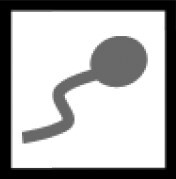 8 months	9–20	Fayomi *et al.* (2019)
		Subcutaneously (scrotum)	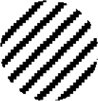 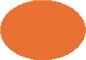 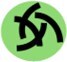 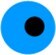 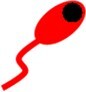 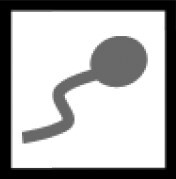 5 months	9–20	Fayomi *et al.* (2019)
**Human**	Pre-pubertal	Subcutaneously (scrotum)	Graft survival	4–21	Goossens *et al.* (2026)
		In the testis	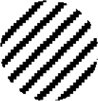 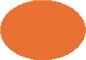 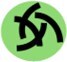 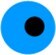 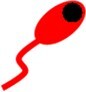 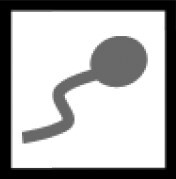 12 months	1–20	Goossens *et al.* (2026)
	Adult	Subcutaneously (scrotum)	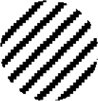 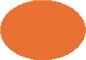 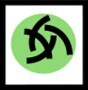 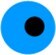 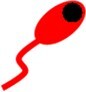 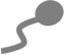 6 months	16–18	Jensen *et al.* (2024)

Hatched areas indicate graft degeneration, orange represents spermatogonia, green represents spermatocytes, blue represents round spermatids, red represents elongating spermatids, and grey represents spermatozoa. The most advanced germ cell stage is outlined. NK, not known.

### Trout

Hayashi *et al.* (2018) demonstrated that frozen-thawed pre-pubertal testicular tissue fragments from rainbow trout can be successfully grafted. If transplanted under the back skin of sexually mature healthy fish, the fragments underwent complete differentiation and produced functional sperm within 5 months post-grafting.

### Rat

Fragments of frozen-thawed rat pre-pubertal testicular tissue were grafted under the scrotal skin. Spermatogonia persisted for at least 1 month after grafting, but this was not followed up further. Interestingly, the number of spermatogonia per tubule in large fragments (27 mm^3^) was significantly lower than in smaller ones (1–18 mm^3^) (Wang *et al.*, 2022).

### Non-human primates

#### Marmoset

Pre-pubertal marmosets were castrated, and their testicular tissue, either fresh or frozen-thawed, was autotransplanted subcutaneously under the back skin while they were still pre-pubertal (Wistuba *et al.*, 2006; Luetjens *et al.*, 2008). After transplantation of fresh grafts, serum testosterone levels remained below the normal range (Wistuba *et al.*, 2006). These grafts were still arrested at the meiotic stage by 17 months post-grafting (Wistuba *et al.*, 2006; Luetjens *et al.*, 2008). Furthermore, Luetjens *et al.* (2008) performed autologous transplantation of pre-pubertal marmoset testicular tissue fragments, either fresh or frozen-thawed, under the scrotal skin, with fresh grafts progressing to complete spermatogenesis.

Ectopic transplantation of adult marmoset testicular tissue resulted in graft degeneration (Luetjens *et al.*, 2008).

#### Rhesus monkey

Testicular tissue fragments from pre-pubertal rhesus monkeys, either fresh or frozen-thawed, were grafted under the shoulder, back, and scrotal skin (Jahnukainen *et al.*, 2012; Fayomi *et al.*, 2019). Autografts transplanted under the shoulder skin failed to mature beyond the pachytene spermatocyte stage after 5 months post-grafting (Jahnukainen *et al.*, 2012); however, spermatogenesis would not be expected to occur within such a short period. Between 8 and 12 months post-grafting, both back skin and scrotal grafts produced testosterone, and FSH levels remained within the normal range, indicating a functional HPG axis capable of supporting endocrine activity. Within the same time frame, complete spermatogenesis was observed in autografts transplanted under the back skin (Fayomi *et al.*, 2019). In scrotal grafts, spermatogenesis established faster, from 5 months onwards (Jahnukainen *et al.*, 2012; Fayomi *et al.*, 2019). Fayomi *et al.* (2019) showed that spermatozoa originating from these scrotal and ectopic autografts successfully fertilized rhesus oocytes, leading to the birth of a healthy female baby, demonstrating the reproductive competence of sperm produced in these autografts. These findings establish a clear proof-of-concept and have contributed to the initiation of a clinical trial.

### Human

A 31-year-old man with idiopathic non-obstructive azoospermia underwent micro-TESE. During this procedure, both small testicular biopsies and spermatozoa retrieved from dilated tubules were cryopreserved, although the total yield was low. Due to the limited number of retrieved spermatozoa, IVF was required. Nevertheless, the resulting embryos did not lead to a successful pregnancy. Approximately 2 years later, the thawed testicular tissue fragments were transplanted into surgically created subcutaneous pockets under the scrotal skin. Six months post-transplantation, graft survival was confirmed, with intact tubules and normal cellular organization. However, spermatogonia and primary spermatocytes were the most advanced germ cells present. No spermatozoa were produced, likely due to the pre-existing spermatogenic failure of the patient (Jensen *et al.*, 2024).

In December 2024, the world’s first autologous grafting of cryopreserved pre-pubertal testicular tissue was performed at the University Hospital of the Vrije Universiteit Brussel. The patient was diagnosed with sickle cell disease and underwent hydroxyurea treatment for 3 years. In 2008, at the age of 10 years, the patient banked testicular tissue prior to starting chemotherapy with busulfan and cyclophosphamide (cyclophosphamide equivalent dose > 5 g/m^2^) for bone marrow transplantation. One entire testis was surgically removed, fragmented, and cryopreserved. At the time of banking, the tissue showed good tubular integrity but contained very few spermatogonia. The patient underwent normal puberty, but was diagnosed with azoospermia at the age of 24 years and again at the age of 26 years. Spermatogonia were absent in the testicular tissue retrieved at the time of transplantation. Fragment sizes ranging from 1 to 21 mm³ were transplanted in the testis and under the scrotal skin. The patient recovered well and did not experience pain or swelling. One year after grafting, the testicular and scrotal grafts were surgically removed and examined for the presence of spermatozoa. SSCs, together with evidence of active spermatogenesis, were identified in two of the four intra-testicular grafts, whereas no germ cells were detected in the subcutaneous grafts (Goossens *et al.*, 2026).

## Discussion

This review demonstrates that the success of testicular tissue transplantation is influenced by multiple factors such as donor age, graft site, fragment size, fresh or cryopreserved grafts, duration of grafting, recipients’ reproductive status, and species-specific characteristics.

Pre-pubertal testicular tissue offers the highest graft survival and functional outcomes in allo-, xeno-, and autotransplantation. Pubertal testicular tissue can also support spermatogenesis, although degeneration was observed over time following xenotransplantation of pubertal dog testicular tissue. In contrast, allo-, xeno-, and autotransplantation of adult testicular tissue generally results in extensive degeneration of the seminiferous tubules and an absence of complete spermatogenesis, except in a few cases, including allotransplantation of adult medaka, xenotransplantation of adult horse, and autotransplantation of adult human testicular tissue. The contrasting outcomes of xenografting pre-pubertal versus adult testicular tissue may be attributed to three main factors. First, steroidogenesis is lower in grafts from adult donors than in those from pre-pubertal donors. This difference is partly due to the ability of pre-pubertal Leydig cells to maintain steroidogenic activity with minimal LH stimulation. In contrast, adult Leydig cells are highly dependent on LH, and transient disruption of this hormonal feedback following transplantation results in a rapid decline in steroidogenesis (Mendis-Handagama and Ariyaratne, 2001). Second, pre-pubertal testicular tissue contains undifferentiated Leydig cell precursors capable of proliferation and regeneration after damage, whereas adult Leydig cells are terminally differentiated and lack this regeneration capacity (Senge *et al.*, 1978; Chen *et al.*, 2010). As a result, grafts from pre-pubertal donors can provide normal to elevated testosterone levels in recipients, while grafts from adult donors may not achieve comparable testosterone secretion (Schlatt *et al.*, 2006). Third, pre-pubertal testicular tissue may better survive oxygen deprivation during the ischaemic period immediately after grafting. Compared to adult tissue, which has a high oxygen demand, pre-pubertal tissue consumes less oxygen and promotes angiogenesis more effectively after transplantation (Devi *et al.*, 2017). Furthermore, foetal testicular tissue rarely achieves complete spermatogenesis. This may be explained by the fact that Sertoli cells have not yet fully differentiated and lack functional maturation (Yokonishi and Capel, 2021). These findings indicate that the developmental stage of donor testicular tissue influences graft survival, germ cell differentiation, and long-term function, with pre-pubertal testicular tissue appearing as the most promising and clinically viable option. In the clinic, pre-pubertal testicular tissue can thus be preserved and used to restore fertility at adulthood.

The site of grafting plays a crucial role in the outcome of testicular tissue transplantation. Homotopic engraftment generally provides the most consistent support to restore spermatogenesis. In contrast, ectopic grafts yield variable outcomes, with complete spermatogenesis not consistently observed. Therefore, homotopic grafting is the preferred approach for clinical application.

Testicular tissue fragments used for transplantation vary widely in size, typically ranging from ∼0.5 to 2 mm³ in most studies to larger pieces of up to 20 mm³. Overall, complete spermatogenesis can be achieved across this size range, although very large fragments (e.g. 27 mm³) may contain significantly fewer spermatogonia per tubule compared to smaller ones.

No notable differences in graft survival or developmental outcomes were observed between fresh and cryopreserved grafts, supporting the feasibility of using cryopreserved tissue in clinical applications.

It has also been shown that the functional lifespan of the grafts is limited, with spermatogenic activity declining over time. This highlights the need to determine the optimal grafting duration.

The recipients’ reproductive status had no effect on graft success, with complete spermatogenesis observed across all recipients, including non-castrated animals, animals subjected to sexual abstinence, animals with induced abdominal cryptorchidism, and partially or fully castrated animals.

After homotopic xenotransplantation, it was shown that the donor-host relationship between mouse and human affects the outcome of human testicular tissue transplantation. In this case, hormonal incompatibilities occur due to the inability of murine gonadotrophins to effectively activate human receptors, thereby impairing testicular cell maturation and spermatogenesis (Hutka *et al.*, 2020). However, in a clinical setting, autotransplantation is the only accepted approach to directly assess the capacity of human testicular tissue to restore complete spermatogenesis.

Although testicular tissue transplantation shows promising outcomes, the risk of reintroducing malignant cells warrants careful evaluation, as cryopreserved testicular tissue from boys with haematological malignancies may contain malignant cells (Kourta *et al.*, 2024). As investigated in rats, the reintroduction of only 20 leukaemic cells is sufficient to cause a cancer relapse (Jahnukainen *et al.*, 2001). Nevertheless, transplantation of testicular tissue from patients with a history of solid-non-metastatic malignancies is associated with a lower risk of malignant cell reintroduction because the tumour remains localized (Safrai *et al.*, 2025). However, thorough risk assessment is essential to ensure the safety of cryopreserved testicular tissue, using a combination of multiple complementary diagnostic methods. For example, histological analysis enables the identification of abnormal cellular morphology and features suggestive of malignancy, while immunohistochemical (IHC) analysis allows detection of specific tumour markers, thereby increasing the specificity for identifying malignant cells. In addition, PCR offers highly sensitive detection of molecular markers and reveals minimal residual disease that could be missed by IHC. However, since the analysis will be done on a fragment of the tissue that will no longer be useful for transplantation, no diagnostic method is able to guarantee that the fragment for transplantation is entirely free of malignant cells (Kourta *et al.*, 2023). Therefore, if there is a risk of malignant cell reintroduction, testicular tissue transplantation is not recommended, and SSCT represents a safer approach. Two main strategies have been investigated to prevent malignant cell reintroduction during SSCT. The first strategy is culture-based purging, in which testicular cell suspensions are cultured under conditions that do not support malignant cell survival. The second strategy is marker-based selection, which removes malignant cells or isolates SSCs using cell surface markers. Despite extensive research, these strategies have not yet been translated into clinically applicable methods.

To conclude, the most promising results were obtained after allo-, xeno-, and autotransplantation of pre-pubertal testicular tissue, with homotopic engraftment being the preferred graft location. Moreover, cryopreservation preserves grafting efficiency, with no differences observed compared to fresh grafts. Nevertheless, the functional lifespan of the grafts may be limited, underscoring the need to identifying the optimal grafting duration.

These pre-clinical findings have supported the clinical translation of autologous transplantation of human pre-pubertal testicular tissue, which is currently being evaluated in the first clinical trials.

## Data Availability

No new data were generated or analysed in support of this review.
